# Dermal T cell immunity and key regulatory signaling pathways: Implications in immune-mediated alopecia and hair regeneration

**DOI:** 10.1016/j.gendis.2025.101518

**Published:** 2025-01-08

**Authors:** Nana Tao, Qingru Sun, Yuyuan Ying, Yitao Wang, Jianli Gao

**Affiliations:** a School of Pharmaceutical Sciences, Zhejiang Chinese Medical University, Hangzhou, Zhejiang 310053, China; b State Key Laboratory of Quality Research in Chinese Medicine, University of Macau, Macao 999078, China; c Clinical Research Institute, Zhejiang Provincial People's Hospital (Affiliated People's Hospital), Hangzhou Medical College, Hangzhou, Zhejiang 310014, China

**Keywords:** Autoimmunity, Hair, Hair follicle regeneration, Immune homeostasis, T cell

## Abstract

Mammalian hair follicles undergo periodic regeneration, with recent research highlighting the immunological niche as a critical regulator of stem cell activity and hair follicle regeneration. Chemotactic signals from hair follicles attract macrophages and T cells, which, in turn, control the resting and differentiation of epithelial stem cells in both healthy and damaged conditions. T cells play a pivotal role in hair follicle regeneration, contributing to injury-induced hair neogenesis and physiologic hair cycling. However, disruption of this interaction can lead to clinically significant immune-mediated alopecia. Both scarring and non-scarring forms of alopecia arise from an imbalance in this dynamic system. In this review, we address the role of T cells in hair follicles, summarize related mechanisms, and highlight key genes involved in T cell differentiation and development. Our aim is to provide insights into the development of hair disorders linked to T cell immune homeostasis and hair follicle regeneration.

## Introduction

**Figure 1:**
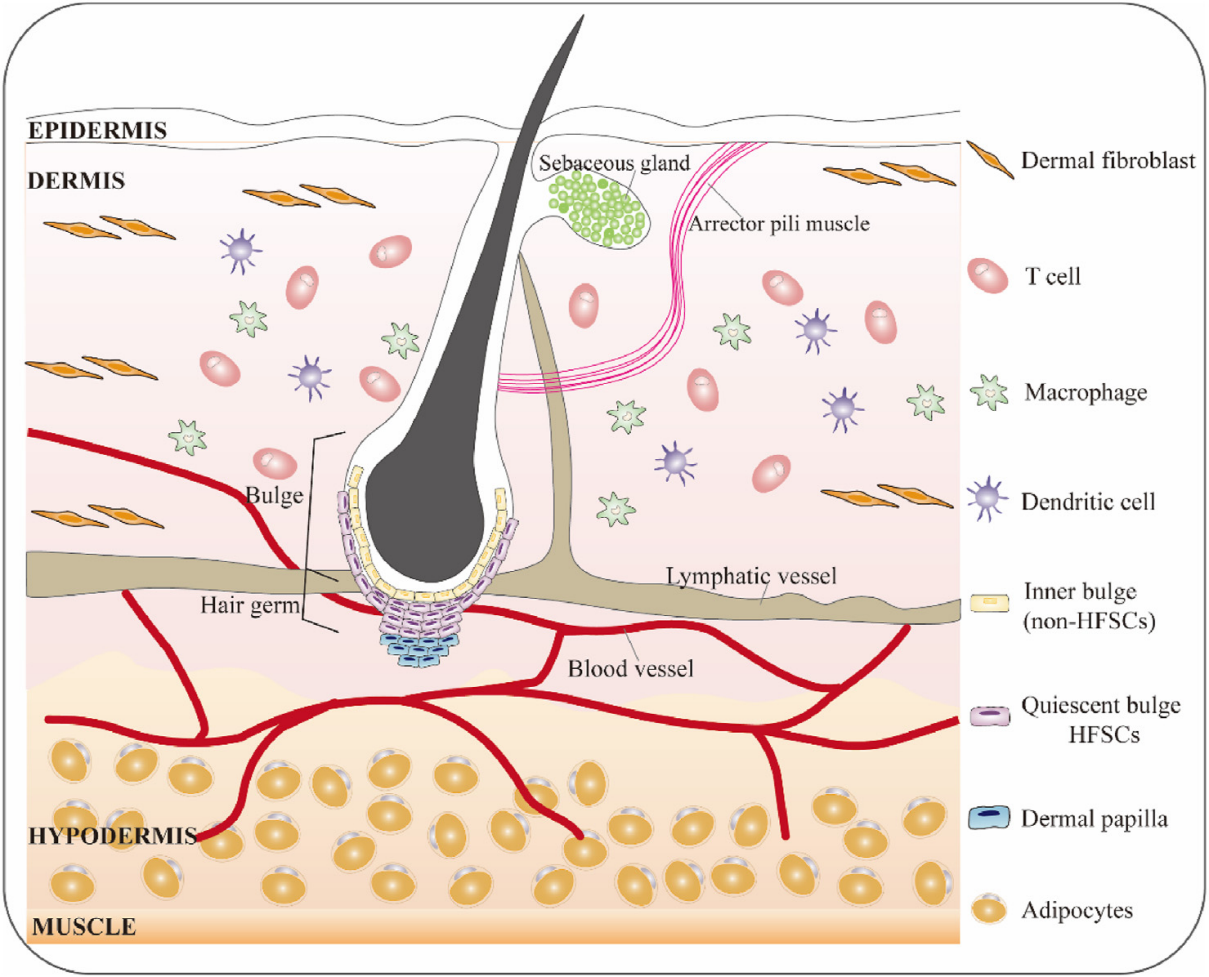
The basic structure of HFSC and components of its niche. Around HFs, there is usually a microenvironment of HFSCs composed of dermal fibroblasts, immune cells, adipocytes, and other cell types. HF, hair follicle; HFSC, hair follicle stem cell.

The skin is a well-structured, layered tissue composed of the epidermis, dermis, and subcutaneous tissue. Hair follicles (HFs) are skin appendages that extend downward from the epidermis into the dermis, originating from the ectoderm. The morphogenesis and development of HFs occur in three stages: induction (generation of hair substrate), organogenesis (downward growth of hair substrate), and cell differentiation (morphogenesis of HFs).^1^ The growth of normal HFs follows a cyclical pattern, progressing through the anagen, catagen, and telogen phases (Fig. 1).^2^ Serving as a stem cell reservoir and hair shaft producer, HFs play a role in shaping the surrounding skin environment, including its innervation and vascularization.^3^ Hair follicles perform various functions, such as physical protection, sensory perception, thermal insulation, sebum distribution, and social signaling. Additionally, hair holds significant cultural and personal value, impacting quality of life, beauty, and self-esteem. However, inflammation caused by various factors can lead to permanent hair follicle loss, compromising both skin function and psychological well-being. This has driven a growing interest in HF regeneration, which presents favorable market potential.^4^ HFs, however, are influenced by a complex interaction of chemical signals and cellular processes.

In the mid-20th century, discoveries led to the development of trichoimmunology, a field that investigates the immune system's role in HFs. This spurred research into immune privilege (IP), immune-mediated alopecia, and the maintenance of immune tolerance.^5^ Billingham et al demonstrated that the epithelial bulb of the hair follicle is one of the few immune-privileged sites in the mammalian body.^6^ Mammalian skin harbors a large population of T cells, which are vital for protecting against infection and maintaining skin barrier integrity and tissue balance.^7^ Dysfunction in these T cells is a hallmark of several skin conditions.^8^ Disruptions in the immunological microenvironment can impair the hair follicle cycle and IP, ultimately leading to HF fibrosis and hair loss.^9^ Therefore, regulating T cell activity may offer a promising therapeutic avenue for HF disorders.^8^^,^^10^

In this review, we explore the role of T cell dysregulation in HFs, summarize the mechanisms involved, and highlight key genes that influence T cell differentiation and development.

## The immune-mediated diseases of hair

**Figure 2:**
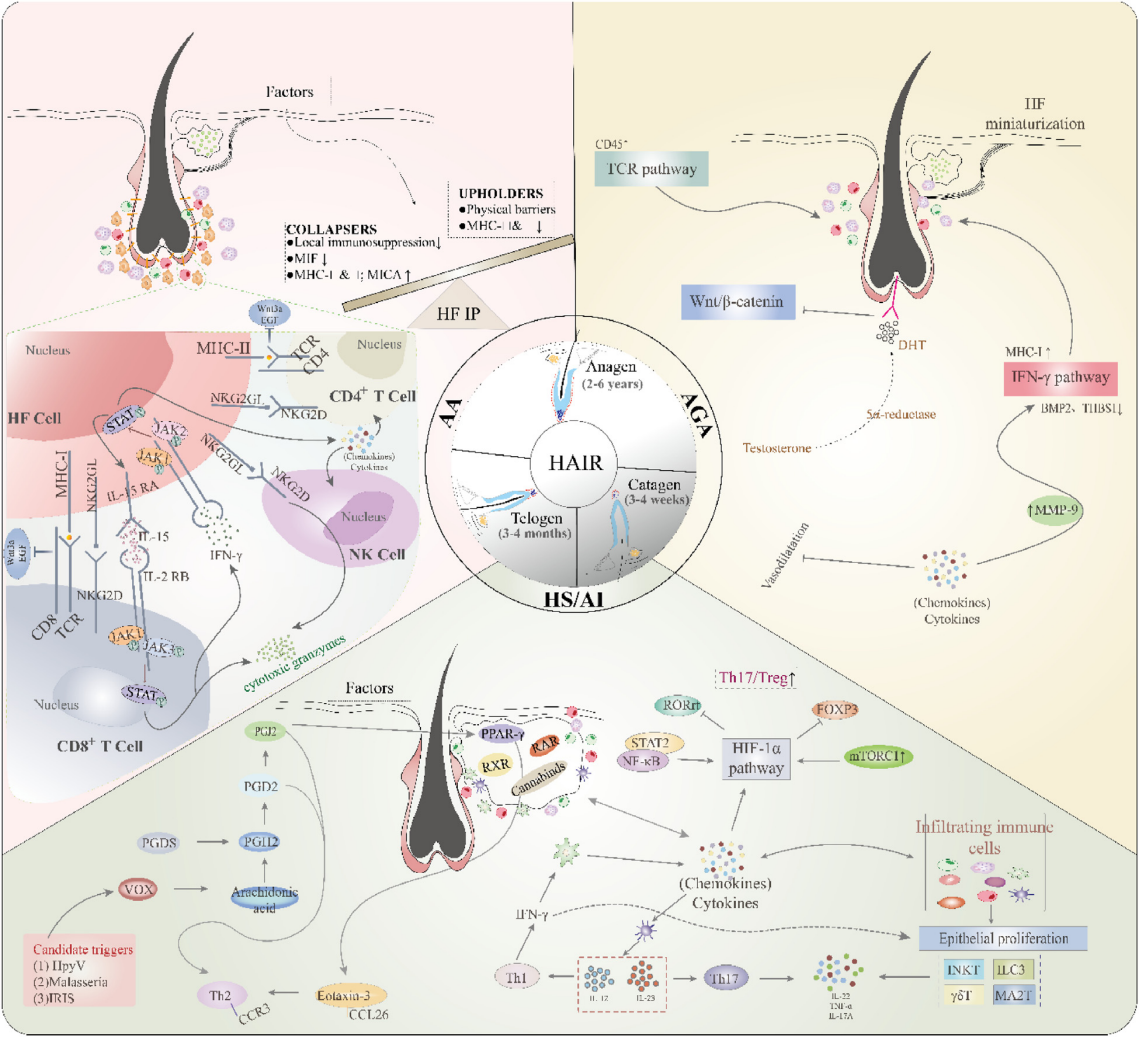
Immunological aspects of alopecia subtypes. HF growth goes through the anagen, catagen, and telogen phases. Disruption of the dynamic relationship between HFs and the surrounding immune microenvironment often results in the breakdown of the HF cycle, leading to alopecia. HF, hair follicle; HS, hidradenitis suppurativa; AI, acne inversa; AA, alopecia areata; AGA, androgenetic alopecia; IP, immune privilege; TCR, T cell receptor; NK, natural killer; MHC, major histocompatibility complex; MMP-9, matrix metallopeptidase 9; IL, interleukin; IFN-γ, interferon-γ; Th, T helper cell; DHT, dihydrotestosterone; BMP, bone morphogenetic protein; MMP, matrix metallopeptidase; HIF-1α, hypoxia-inducible factor-1α.

The normal hair cycle relies on continuous interactions between HFs and their immunological environment. Specific components of HFs, such as protrusions during the hair cycle and bulbs in the growth phase, help maintain a degree of IP. This relationship between HFs and the immune system is sustained by the IP mechanism. In a healthy scalp, the hair bulb and bulge show reduced or absent expression of major histocompatibility complex (MHC) molecules, which limits the display of self-antigens to CD4^+^ and CD8^+^ T cells. Immunosuppressive molecules, such as transforming growth factor (TGF)-β1/2, interleukin (IL)-10, α-melanocyte-stimulating hormone, and vasoactive intestinal peptide, inhibit antigen-presenting cells, leading to lower levels of activated T cells. Furthermore, the immunosuppressive environment decreases the presence of natural activators (*e.g.*, MICA and ULBP3) for NKG2D (natural killer group 2 member D)-expressing cells, including natural killer cells and γδ T cells.^10^ HFs regulate epithelial cell behaviors, including resting, growth, and differentiation, through chemotaxis and recruitment of T lymphocytes under both normal and damaged conditions. Disruption of this dynamic connection can result in significant hair loss. Excessive immune cell infiltration may lead to hair loss by negatively affecting HF stem cells (HFSCs) and compromising the circulation within HFs.^9^ In summary, when HF immune privilege (HF-IP) collapses, inflammatory cells infiltrate the bumps and bulbs of HFs, triggering an immune response that leads to autoimmune diseases and hair loss (Fig. 2).^11^

## Alopecia areata

Alopecia areata (AA) is a complex condition characterized by non-scarring and reversible hair loss in susceptible individuals, with its progression influenced by various factors and often following an unpredictable course.^12^ AA is primarily an autoimmune disorder involving T lymphocytes.^13^ Based on its severity, AA can be classified into three forms: patchy AA, alopecia totalis, and alopecia universalis. The immune responses associated with AA are typical of autoimmune diseases, including local inflammation, detection of autoantigens, a cytokine/chemokine storm, loss of HF-IP, and immune cell infiltration.^14^ The primary theory behind its pathogenesis is that AA arises from the breakdown of HF-IP. AA is characterized by the presence of lymphocytes surrounding HFs, with CD8^+^ T cells found in the follicular epithelium and CD4^+^ T cells around the follicles. The collapse of HF-IP is thought to trigger AA.^15^ When HF-IP fails, NKG2D^+^ natural killer cells, along with self-reactive NKG2D^+^ CD8^+^ T cells, recognize autoantigens and attack the HFs. This loss of IP leads to increased cytokine production, including interferon-γ (IFN-γ), and heightened expression of MHC-Ia.^15^ The up-regulation of MHC expression correlates with increased mast cell maturation, eosinophil influx, and the accumulation of CD8^+^ T cells, CD4^+^ T cells, natural killer T cells, peripheral dendritic cells, and Langerhans cells. Additionally, serum cytokine profiles show elevated levels of proinflammatory cytokines associated with T helper cell type 1 (Th1), Th2, and Th17, along with an increase in IFN-γ in the blood (see Table 1).^16^

**Table 1:** Comparison of the immune-mediated alopecias.

Immune-mediated alopecia subtypes		Immune aspects (pathogenesis)	Clinical features and histopathology
Non-scarring alopecias	AA	1. The collapse of HF-IP; 2. Up-regulation of MHC class I; 3. Dense accumulation of lymphocytes (CD4^+^ T, CD8^+^ T, natural killer cells) around the hair bulb; 4. Increased IFN-γ expression and chemokines CXCL9/MIG and CXCL10/IP-10; 5. Increase in CXCR3^+^ CD4^+^ Th1 and CXCR3^+^ CD8^+^ Tc1 cells; 6. Elevated NKG2D expression in CD8^+^ T cells and CD56^+^ natural killer cells; 7. Involvement of Th17 cells (increased IFN-γ, IL-17, IL-4, IL-10); 8. Increased TLR7 and TLR9 expression.	1. Hair loss pattern: Single delimited patches, patchy AA, alopecia totalis, alopecia universalis, reticular patches of hair loss; 2. Monocyte accumulation around the hair bulb; 3. HF miniaturization with reduced cell accumulation; 4. Complications: Lupus erythematosus, vitiligo, psoriasis.
Non-scarring alopecias	AGA	1. The collapse of HF-IP; 2. Up-regulation of MHC class I; 3. Excessive DHT-androgen receptor binding causes immune cell infiltration (central memory CD8^+^ T cells, CD4^+^T cells, γδ T cells, mast cells) in the HF bulge; 4. Up-regulation of MMP9 and CD45; 5. Down-regulation of BMP2 and THBS1; 6. Up-regulation of cytokines (IFN-γ, EGF, TNF-α, IL-1α, TGF-β).	1. Pattern: Receding hairline and diffuse hair loss; 2. Fibrosis occurs in the HF bulge, associated with HF miniaturization.
Scarring alopecias	LPP	1. The collapse of HF-IP; 2. Up-regulation of MHC class I and II; 3. Down-regulation of CD200; 4. Increased expression of IFN-inducible cytokines (CXCL9/CXCL10/CXCL11); 5. CD8^+^ T cell infiltration predominates.	1. T-cell-mediated follicular inflammatory infiltrates in the infundibulum and isthmus near the HF bulge; 2. Irreversible and permanent alopecia; 3. Variants: Classic form, FFA, Graham-little syndrome; 4. Follicular hyperkeratosis, perifollicular erythema, and scaling.
Scarring alopecias	FFA	1. The collapse of HF-IP; 2. Increase in CD8^+^ T cells and dendritic cells; 3. Down-regulation of TGF-β2, PPAR-γ, and CD200; 4. Up-regulation of IFN-γ, MHC-I, MHC-II, and β2-microglobulin.	1. Receding hairline in the frontal and temporal lobes; 2. Inflammatory cell infiltration near the bulge and funnel.
Scarring alopecias	CCCA	1. PADI3 gene mutation; 2. CD4^+^ T cell infiltration predominates; 3. CD3 and CD4 T-lymphocytes in both affected and unaffected follicles; 4. Increased CD1a ratio.	1. Pattern: Patchy scarring lesions, starting at the posterior crown or vertex and extending centrifugally; 2. Premature desquamation of the inner root sheath; 3. Lamellar hyperkeratosis or parakeratosis; 4. Naked hair shafts in the dermis; 5. Follicular miniaturization.
Scarring alopecias	HS/AI	1. Neutrophil and lymphocyte infiltration; 2. Perifollicular immune cell infiltration; 3. Up-regulation of TNF-α, IL1β, IL-17, and IFN-γ; 4. Imbalance of Th17 cells (increase), Tregs (decrease), and Th1 cells; 5. Up-regulation of HIF-1α; 6. Notch signaling disorder.	1. Pattern: Patchy scarring hair loss with pustular lesions, primarily affecting men; 2. Excess keratin production and epidermal hyperplasia of the follicular infundibulum; 3. Follicular occlusion followed by dilation and stasis of the HF.

Note: HF, hair follicle; HF, hair follicle immune privilege; AA, alopecia areata; AGA, androgenetic alopecia; LLP, lichen planopilaris; FFA, frontal fibrosing alopecia; CCCA, central centrifugal cicatricial alopecia; HS, hidradenitis suppurativa; AI, acne inversa; MHC, major histocompatibility complex; IFN-γ, interferon-γ; TLR7/9, Toll-like receptor 7/9; IP, immune privilege; DHT, dihydrotestosterone; BMP2, bone morphogenetic protein 2; THBS1, thrombospondin 1; IL, interleukin; Th, T helper cell; TNF-α, tumor necrosis factor-α; Tc cell, conventional T cell; TGF-β, transforming growth factor beta; NKG2D, natural killer group 2 member D; EGF, epidermal growth factor; PPARγ, peroxisome proliferator-activated receptor γ; PADI3, peptidyl arginine deiminase 3.

In the initial phase of AA, CD8^+^ T cells play a key role by driving an inflammatory cycle that causes damage to HFs.^17^ Once activated, CD8^+^ T cells secrete IFN-γ, which interacts with receptors on follicular epithelial cells, initiating a signaling cascade that includes JAK1/JAK2 signaling transducers. These transducers regulate IL-15 production, which binds to the IL-2/IL-15Rβ receptor complex, connected to JAK1/JAK3 signaling on CD8^+^ T cells. This activation stimulates CD8^+^ T cell proliferation and inflammatory responses.^18^

In addition to CD8^+^ T cells, regulatory T cells (Tregs) are also implicated in the pathogenesis of AA. Recent evidence suggests that a deficiency of Tregs is an influencing factor in AA. Damaged Tregs in HFs contribute to local immune dysregulation and hinder HF regeneration.^13^

A recent study has also suggested that the Th2 pathway may play a role in AA. A genome-wide association study identified an IL-13 susceptibility locus for AA. Seventeen patients with AA exhibited both Th1 and Th2 immune skewing in blood and scalp biopsies. Furthermore, total serum immunoglobulin E (IgE) levels were elevated in AA patients, regardless of additional atopic conditions.^19^ Biomarker analysis of JAK1/TYK2 and JAK3/TEC dual inhibitors showed that both Th2 and Th1 biomarkers were linked to therapeutic efficacy.^20^ The detrimental role of the Th2 pathway in AA was further confirmed in a clinical study using dupilumab, a drug targeting IL-4Rα. Additional research found that HFs were initially associated with a decrease in Th2 inflammation and an increase in hair keratin genes, followed by a reduction in Th1 activity, suggesting a pathogenic role for the Th2 axis in AA. Other cytokines implicated in AA include IL-9, IL-23, and IL-32.^18^ Notably, unlike cicatricial alopecia, AA primarily affects the hair bulb. In acute AA, immune cells infiltrate the bulb and bulge regions of HFs, which may explain why HFs in AA patients often retain the ability to regenerate.^21^^,^^22^

## Androgenetic alopecia

Androgenetic alopecia (AGA) is characterized by a gradual pattern of hair loss, typically beginning with the receding of the bilateral temporal lobes at the front hairline, followed by overall thinning at the crown of the head.^23^ In individuals with AGA, elevated levels of 5α-reductase lead to the conversion of testosterone into dihydrotestosterone within cells. Increased levels of dihydrotestosterone bind more strongly to androgen receptors, which results in the suppression of scalp hair growth and the shrinking of HFs.^24^ During a typical HF cycle, CD8^+^ T cells and γδ T cells are present at their lowest levels around the HFs during the telogen (resting) phase, and peak during the anagen (growth) phase.^25^ While AGA was once considered a non-inflammatory and non-scarring form of alopecia, recent studies have revealed the presence of micro-inflammatory responses in affected individuals. Earlier research identified mononuclear cells and lymphocytes in approximately 50 % of male AGA scalp samples, with evidence of mast cell granules and fibroblast activation in the fibrous sheath.^26^ Activated T cells have also been detected in the lower portions of the follicular infundibula.^27^ The imbalance in immune cell infiltration in AGA may be linked to the excessive binding of dihydrotestosterone to androgen receptors, which suppresses hair growth and leads to HF miniaturization.^9^ This overabundance of dihydrotestosterone-androgen receptor binding triggers increased expression of pro-inflammatory molecules like tumor necrosis factor-α (TNF-α) and IL-1α while reducing the expression of bone morphogenetic protein 2 (BMP2) and thrombospondin 1 (THBS1). This, in turn, stimulates the production of matrix metallopeptidase 9 (MMP-9), which interferes with the IFN-γ signaling pathway, up-regulating MHC-I expression in HF-IP. The collapse of HF-IP promotes CD8^+^ T cell attacks on HFs, leading to immune cell infiltration in the HF bulge area. Additionally, the protein tyrosine phosphatase receptor type C, or CD45, is a key player in transmitting signals in T cell receptor pathways. Studies have shown that elevated CD45 levels are associated with increased CD8^+^ T cells and CD4^+^ T cells around HFs in individuals with AGA.^9^^,^^28^, ^29^, ^30^

## Lichen planopilaris

Lichen planopilaris (LPP) is an inflammatory condition that causes scarring hair loss, predominantly affecting perimenopausal or postmenopausal women.^31^ LPP is a form of primary cicatricial alopecia, characterized by lymphocytic infiltration and typically presenting as hair loss on the scalp, particularly at the crown. Patients often report symptoms such as itching, burning, or discomfort on the scalp.^32^ The collapse of HF-IP, induced by IFN-γ, plays a key role in the pathogenesis of LPP.^33^ Additionally, T cell-mediated autoimmune targeting of HF antigens may contribute to HF destruction.^34^^,^^35^ LPP has three recognized subtypes: the classic form, frontal fibrosing alopecia (FFA), and Graham-Little syndrome. All subtypes share similar histological features, with HFs specifically damaged by a prolonged inflammatory process involving lymphocytes, while the interfollicular skin remains unaffected.^36^ Research has identified the presence of activated T cells surrounding HFs in LPP patients, particularly in the bulb and bulge areas, characterized by increased CD8^+^ T cells and dendritic cells resembling plasma cells.^37^^,^^38^

Usually, the typical ratio of CD4^+^ T to CD8^+^ T cells in healthy HFs is 2:1. However, in advanced lesions of LPP, CD8^+^ T cells become predominant, altering the CD4^+^ T cells ratio to 1:1, while the number of Langerhans cells decreases. CD8^+^ T cells play a regulatory role in the inflammatory response, contributing to pathogenic changes in the HFs.^22^^,^^33^ LPP is also marked by elevated levels of CD8^+^ granzyme B^+^ T cells and plasmacytoid dendritic cells, along with increased expression of the chemokine receptor CXCR3.^33^ The lesions in LPP typically exhibit a band-like subepidermal lymphocytic infiltrate around the upper HFs, specifically in the isthmus and infundibulum, while the deeper parts of the follicle remain unaffected, as seen in AA. In later stages, LPP is characterized by a reduction or loss of sebaceous glands and arrector pili muscles, centripetal fibrosis around HFs, irreversible destruction of HFs, and glassy degeneration in the upper and lower dermis and HF ducts.^34^

## Frontal fibrosing alopecia

Frontal fibrosing alopecia (FFA) is a form of primary cicatricial alopecia caused by lymphocytic infiltration, leading to permanent hair loss. It is characterized by a receding hairline in the frontal and temporal regions and is often considered a variant of LPP.^39^ Numerous studies have highlighted the role of immune cell-mediated inflammatory responses in the pathogenesis of FFA.^33^ Histological findings in FFA show that CD8^+^ T cells, plasma cell-like dendritic cells, and Langerhans cells infiltrate the HF bulge area and near the infundibulum.^31^ Compared with normal HFs, FFA exhibits an up-regulated expression of IFN-γ, major histocompatibility complex I (MHC-I), and other proteins, which leads to the collapse of HF-IP and the development of follicular fibrosis. Additionally, dysregulation of the PPAR-γ and mTOR signaling pathways further contributes to the pathogenesis of FFA.^37^

## Central centrifugal cicatricial alopecia

Central centrifugal cicatricial alopecia (CCCA) is a type of scarring hair loss that predominantly affects women of African heritage.^40^, ^41^, ^42^ CCCA is characterized by follicular inflammation, follicular degeneration syndrome, and pseudovalve formation.^22^ The key histopathological features of CCCA include: i) absence or reduction of sebaceous glands, ii) premature shedding of the inner root sheath, and iii) follicular shrinkage. In the active stages of CCCA, there is follicular lichenoid infiltration, while in later stages, follicular fibrosis becomes the dominant feature.^41^ Unlike LPP and FFA, CCCA is associated with a CD4-predominant lymphocytic inflammatory infiltrate. Both CD3 and CD4 T lymphocytes are present in affected and unaffected follicles, with a higher proportion of CD4^+^ T cells in affected follicles and an elevated CD1a:CD3 ratio.^42^ Furthermore, increased infiltration of Langerhans cells has also been linked to CCCA.^40^ Recent research has shown elevated nuclear phospho-STAT3 levels in perifollicular lymphocytes, indicating that Th17 cells may play a role in the pathogenesis of CCCA.^43^

## Hidradenitis suppurativa/acne inversa

Hidradenitis suppurativa (HS) is a chronic inflammatory skin disorder, also known as acne inversa or “dissecting terminal hair folliculitis” due to its distinctive characteristics.^44^^,^^45^ Elevated levels of proinflammatory cytokines such as TNF-α, IL-1β, IL-17, and IFN-γ have been shown to contribute to immune dysfunction in HS.^46^^,^^47^ Notably, increased activation of hypoxia-inducible factor-1α (HIF-1α), a downstream effector of the rapamycin complex 1 (mTORC1), has been observed in the skin of individuals with HS, suggesting a potential link between metabolic and immune abnormalities in this condition. On one hand, HIF-1α stimulates the formation of Th17 cells by activating the transcription of retinoic acid-related orphan receptor γt (RORγt), a key transcription factor for Th17 cell differentiation. On the other hand, HIF-1α may inhibit the development and function of Tregs by interacting with Foxp3 and promoting its degradation.^45^ Autoimmune diseases associated with HS are primarily linked to an imbalance in the Th17/Treg axis, with Th17 cells being the predominant immune state.^48^

## T cells that play a role in hair regeneration

**Figure 3:**
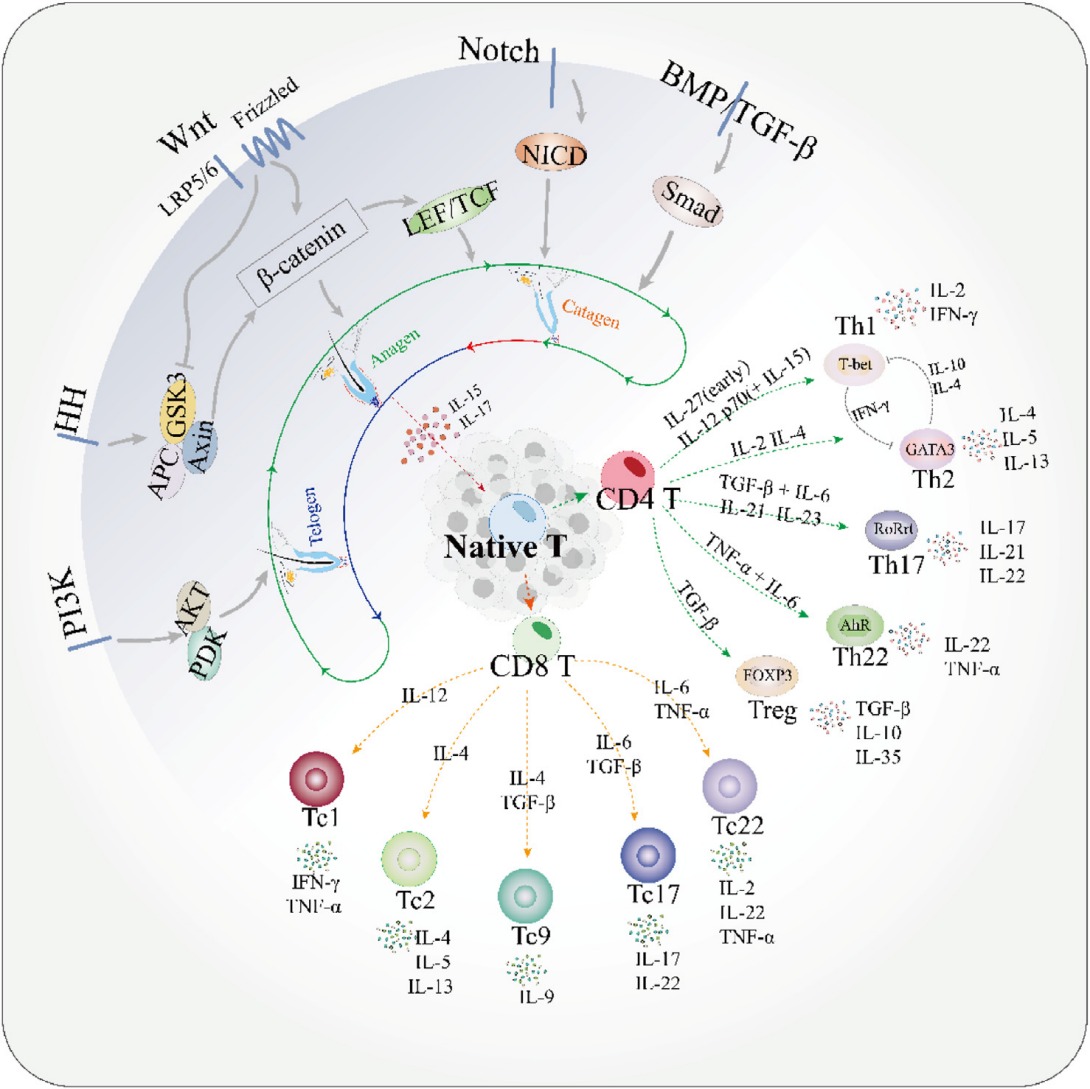
Interaction of signal pathways in HF stem cells and mechanism of T cell differentiation and development. HF, hair follicle; IL, interleukin; APC, antigen-presenting cell; IFN-γ, interferon-γ; Th1, T helper cell type 1; Th2, T helper cell type 2; Th9, T helper cell type 9; Th, T helper cell; TNF-α, tumor necrosis factor-α; BMP, bone morphogenetic protein; Tc cell, conventional T cell; TGF-β, transforming growth factor beta; LRP, low-density lipoprotein receptor-related protein.

The immune system plays a key role in promoting HF regeneration. Pro-inflammatory macrophages stimulate the production of mediators like TNF and IL-1β, which are critical for supporting HF regeneration.^49^, ^50^, ^51^ In addition to macrophages, T cells can differentiate into specialized subsets, such as T helper cells, to regulate the skin's local immune environment and drive the immune response through the Th1/Th2/Th17 paradigm.^52^, ^53^, ^54^ For instance, Tregs promote HFs by mitigating the potentially harmful effects of the innate immune system, specifically by inhibiting the activity of macrophages.^55^^,^^56^ T cells undergo gene rearrangement, T cell receptor recognition, and selection to develop into CD4^+^ T cells and CD8^+^ T cells, each playing distinct roles in HF growth and regeneration (Fig. 3).

## CD4^+^ T cells

Upon activation and differentiation into various effector subtypes, CD4^+^ T cells play a crucial role in the immune response by secreting distinct cytokines (Table 2). CD4^+^ T cells perform multiple functions, including stimulating cells of the innate immune system, B lymphocytes, cytotoxic T cells, and non-immune cells, as well as regulating immune suppression. The main CD4^+^ T cell subsets include Th1, Th2, Th9, Th17, Th22, and Treg cells. Unlike Th1 cells, which are typically terminally differentiated, Th2, Th17, and Treg cells exhibit plasticity.^57^ TGF-β can induce Th2 cells to shift their cytokine profile to one based on IL-9, transforming them into Th9 cells.^58^ In the presence of IL-12, Th17 cells can differentiate into a Th1 phenotype, while exposure to IL-4 promotes their transformation into Th2 cells.^58^^,^^59^ Additionally, Tregs have the potential to differentiate into Th17 cells, with IL-6 driving this transformation upon activation.^60^ If interferon regulatory factor 4 (IRF4) is inactivated in Tregs, the development of Th2 cells and the formation of germinal centers are enhanced.^61^

**Table 2:** Different subtypes of T cells and related T cells in hair regeneration.

T cell	Differentiation	Transcription factor		Factors	miRNA	Signal pathway	Changes in HF regeneration
CD4^+^ T	Th1	IL-12, IFN-γ	T-bet, STAT1, STAT4	IFN-γ, TNF-α, IL-2, IL-12, GM-CSF, CXCR3	**↑**miR-21; miR24; miR-10a; miR-17-92; miR-17; miR-19b; **↓**miR-29a; miR-29b; miR-155; miR-146a; miR-125;	TLR7/MyD88/IRAK1/IKKα/IRF7; TLR8/MyD88/TAK1/IKKβ (or JNK); NF-κB; JAK/STAT; p38/MAPK; JNK.	1.Inhibition of HF proliferation; 2.Disruption of the hair growth cycle; 3.Cavitation of stromal cells; 4.Destruction of melanocytes; 5.Abnormal keratosis of the inner root sheath.
CD4^+^ T	Th2	IL-2, IL-4	GATA3, STAT6	IL-4, IL-5, IL-10, IL-13, CCR4	**↑**miR-21; miR-17-92; miR-126; miR-210; **↓**miR-24; miR-27; miR-128; miR-182; miR-125; miR-29a; miR-155.	NF-κB	1.Inhibition of terminal differentiation proteins in keratinocytes; 2.Promotion of HF cell proliferation and differentiation; 3.Disruption of skin barrier function stability.
CD4^+^ T	Th17	IL-6, IL-21, TGF-β, IL-1β, IL-23	RORγt, IRF4, BATF, AHR, STAT3	IL-17, TNF-α, IL-6, IL-21, IL-22, CCR6	**↑**miR-326; miR-301a; miR-183C; miR-10a; miR-873; miR-351; miR-384; miR-146a; miR-155; **↓**miR-125.	CXCL5/IL-17A/neutrophil; TGF-β.	1.Destruction of HF-IP; 2.Impairment of HF regeneration; 3.Disruption of the hair growth cycle.
CD4^+^ T	Th22	IL-6, TNF-α, IL-1β, IL-21, IL-23	AHR	IL-22, TNF-α, IL-13, CCR10	**↑**miR-31; **↓**miR-125; miR-323-3p.	MEK/ERK; JunK; MAPK pathway; NF-κB; p38 pathway, JAK/STAT; Notch.	1.Protective role in skin inflammatory diseases; 2.Promotion of HFs.
CD4^+^ T	Treg	TGF-β, IL-2	Foxp3, STAT5, STAT3,	TGF-β, IL-10, CD25, CTLA4, CD39	**↑**miR-155; miR-21; miR-24; miR-124a; miR-99a; miR-150; miR-15a/16; miR-15b/16; miR-10a; miR-126; miR-17; miR-31; miR-125a-5p; **↓**miR-100; miR-23; miR-27; miR-125.	Jagged 1/Notch; TGF-β; PI3K/AKT/mTOR.	1.Protection of HF-IP; 2.Located in HFSC niche, promoting HF regeneration; 3.Maintenance of self-tolerance and immune homeostasis, promoting immunosuppression; 4.Immunosuppressive properties do not drive HFSC activation.
CD8^+^ T		IL-2, IL-7, IL-12, IL-15, IFN	RUNX3	IFN-γ, IL-2, TNF-α, perforin granzymes	**↑**miR-181a; miR-17-92; miR-146a; miR-150; miR-16; miR-142; miR-15b; miR-150; **↓**miR-15/16.	PI3K/AKT/mTOR; T cell receptor and its signaling pathway.	1.Destruction of HF-IP; 2.Impairment of the hair growth cycle; 3.Destruction of HF cells mediated by Fas or perforin pathways; 4.Indirect damage to HF cells through cytokine release; 5.Ability to easily penetrate intra-follicular regions.

Note: HF, hair follicle; IFN-γ, interferon-γ; IP, immune privilege; IL, interleukin; Th, T helper cell; TNF-α, tumor necrosis factor-α; NF-κB, nuclear factor kappa B; GATA3, GATA binding protein 3; STAT, signal transducer and activator of transcription; RORγt, retinoic acid-related orphan receptor γt; TGF-β, transforming growth factor beta; IRF4, interferon regulatory factor 4; BATF, basic leucine zipper ATF-like transcription factor; AHR, aryl hydrocarbon receptor; CTLA4, cytotoxic T-lymphocyte associated protein 4; CCR10, C–C motif chemokine receptor 10; RUNX3, RUNX family transcription factor 3; PI3K, phosphoinositide 3-kinase; mTOR, mechanistic target of rapamycin; MAPK, mitogen-activated protein kinase; TLR7/8, Toll-like receptor 7/8; HFSC, hair follicle stem cell.

### Tregs

Tregs in the skin are key protectors of HF-IP, creating a localized immunosuppressive environment to maintain HF-IP.^12^ Tregs in the skin are predominantly located around HFs in both humans and mice, playing a crucial role in regulating the self-immunity of HFs, such as in AA.^62^ However, the relationship between tissue-specific stem cells and Tregs is not well understood. Studies suggest that skin Tregs may control the growth and differentiation of HFSCs by activating the Jagged1-Notch signaling pathway, indicating a direct interaction between stem cells and immune cells in the skin.^63^^,^^64^ Tregs in the hematopoietic system can also create immune-privileged areas to protect hematopoietic stem cells from immune attacks.^65^ Similar to the hematopoietic stem cell niche, skin Tregs are specifically located near the HFSC niche. Research by Ali et al analyzed the immune profile of Tregs during different phases of the HF synchronization cycle, finding that Tregs are most concentrated in the dorsal skin during the resting phase of the HF cycle. Deleting Tregs leads to reduced expression of genes involved in HFSC differentiation into the keratinocyte lineage.^66^ This suggests that the stability of HFSCs relies on the presence of activated Tregs in their niche, emphasizing the growing importance of tissue-resident Tregs in stem cell biology.^64^

Additionally, activation of the PI3K-AKT-mTOR axis negatively regulates Tregs, while TGF-β signaling induces Foxp3 expression and histone acetylation and enhances Treg formation. TGF-β2 secreted by Tregs can also activate Smad2/3 in HFSCs, promoting their activation and proliferation.^67^ At the same time, Treg activation and proliferation are largely dependent on IL-2 produced by conventional T cells. In the presence of IL-2, conventional T cells can induce Foxp3 expression in Tregs, while anti-IL-2 antibodies impair Treg function.^13^ Tregs directly influence HF cycling, as shown in AA mouse models. Administering small amounts of IL-2 to individuals with AA has been shown to increase Treg numbers and promote hair regrowth. The absence of Tregs near the hair bulb causes delays in the transition from the last phase of hair growth to the first, reducing HF and HFSC differentiation, and increasing the duration of the final hair growth stage. Moreover, C57BL/6 mice with a double-negative CD80/CD86 phenotype are prone to reduced Tregs, leading to spontaneous AA.^68^ Stimulating myeloid-derived suppressor cell exosomes in an AA mouse model may increase Treg numbers *in vivo*, reducing lymphocyte activity near HFs and promoting hair growth. These findings suggest that Tregs are valuable therapeutic targets.

Notably, Tregs maintain skin health and promote HF regeneration by suppressing inflammation rather than directly activating HFSCs. Inhibiting the INF-γ signaling pathway, which is regulated by Tregs, cannot restore HFSC activation during the initial stages of hair growth induction.^66^ Thus, Treg-mediated immunosuppression is crucial for HFs, and regenerating Tregs will not lead to skin inflammation.^66^

### Th1 cells

Th1 cells are a subtype of effector cells capable of producing IFN-γ, IL-2, and TNF-β. The T-box protein (T-bet) is recognized as the primary transcription factor for Th1 cells. These cells primarily function to eliminate infections from cells and are also associated with organ-specific autoimmune disorders, such as multiple sclerosis.^69^ The cytokines produced by Th1 cells may promote the generation of additional pro-inflammatory cytokines (such as TNF and IL-1α/β), which can hinder the growth of HFs. These cytokines may trigger blood clotting and distortion of the skin, leading to vacuolation of stromal cells, a decrease in matrix size, death of follicular melanocytes, and aberrant keratinization of the follicular bulb and inner root sheath. Studies have shown that Th1 cells play a crucial role in the pathogenesis of AA and other diseases. Analysis of serum from AA patients revealed increased Th1 axis activity, resulting in elevated levels of cytokines (IFN-γ, IL-2, and IL-12) and chemokines (CXCL10, CCL2, and CCL3) in the bloodstream.^70^ Immunohistochemical examination of skin biopsies indicated that CCR5, a cell marker associated with Th1 cells, penetrated both the interior and the region surrounding the medulla oblongata of HFs.^71^

### Th2 cells

Th2 cells primarily enhance the humoral immune response. During HF formation, Th2 cells stimulate B cells by releasing cytokines, including IL-4 and IL-13. Th2 cells can alter the signaling pathways triggered by cytokines like IL-4, which suppress the immunological activity of Th1 and Th17 cells while promoting the growth and differentiation of HFs.^72^ Additionally, Th2 cells may influence HF formation by regulating the generation and function of growth factors, such as fibroblast growth factor and keratinocyte growth factor. The cytokines produced by Th2 cells may impede the synthesis of fully developed proteins in keratinocytes, including filaggrin, loricrin, and involucrin, which compromises the integrity of the skin barrier. Furthermore, the cytokines from Th2 cells have been shown to inhibit the production of antimicrobial peptides in the skin upon stimulation, including β-defensin-2, β-defensin-3, and LL-37 in keratinocytes.^72^

### Th17 cells

Th17 cells produce pro-inflammatory cytokines, including IL-17, IL-22, and IL-23, which are essential for the growth and development of HFs.^57^ The expression of RORγt is crucial for the differentiation of Th17 cells. The Akt-mTORC1 signaling pathway positively regulates Th17 differentiation, primarily relying on HIF-1α.^73^ HIF-1α promotes the formation of Th17 cells by regulating glycolytic activity and enhances their maturation by directly activating RORγt. MicroRNA (miR)-155 plays a vital role in adaptive immune responses, influencing the lineage choice of CD4^+^ T cells.^48^ While Th17 cells are essential for the growth and development of HFs, they are typically not sufficiently cytotoxic to affect the HFs on their own; the presence of CD8^+^ cytotoxic Th1 cells is required for this process.^74^ Th17 cells may initiate damage to the HFs, but CD8^+^ T cells or more potent Th1 cells are needed to continue the attack. IL-17 and Th17 cells are crucial factors in the pathogenesis of alopecia. An excess of Th17 cells can lead to HF damage, with elevated levels of IL-17 found in the blood of individuals with alopecia. These findings suggest that Th17 cells may have a detrimental impact on HFs, hindering hair growth. Additionally, research has demonstrated that miR-155 specifically interacts with the mRNA of suppressor of cytokine signaling 1, a key inhibitor of the JAK/STAT pathway. miR-155 may enhance the development of Th17 cells and the secretion of IL-17A.^75^^,^^76^ Hessam and colleagues have confirmed that miR-155 is significantly expressed in the injured skin of individuals with HS.^77^

### Th22 cells

Th22 cells, a recently identified subset of CD4^+^ T cells, are abundant in human skin and play a significant role in epidermal wound healing.^78^ These cells produce cytokines such as IL-22 and TNF-α, but not IL-4, IL-17, or IFN-γ. Previous research has shown that Th22 cells have a protective function in inflammatory skin conditions and are involved in the progression of several autoimmune disorders.^79^ Although little research has been conducted on the relationship between Th22 cells and HFs, our study suggests that Th22 cells play a crucial role as effector T cells in thymosin β15 (Tβ15)-induced hair regrowth.^80^ Tβ15, a homolog of thymosin β4, enhances HFs by promoting the proliferative activity of HF cells, as demonstrated by our findings.^81^ Moreover, elevated expression of Tβ15 may increase the number of Th22 cells near HFs and accelerate the maturation process of HFs. As expected, the use of an IL-22BP inhibitor disrupted Th22 cell activity and impeded the HF process. These findings suggest that targeting Th22 cells could be a promising strategy for stimulating hair regrowth. Further exploration of the biological role of Th22 cells is anticipated to offer novel insights into the study of hair disorders.

## CD8^+^ T cells

Exposure to antigens or the transfer of activated lymphocytes can trigger autoimmune disorders targeting immune-privileged areas, potentially disrupting HF-IP by inducing the immune system to attack HF autoantigens.^82^ Abnormal expression of CD4^+^ T cells may lead to the collapse of HF-IP, while CD8^+^ T cells are rarely present around the expanding HFs of healthy skin. However, activation of CD8^+^ T cells may result in a more widespread immune response (Table 2).^83^ CD8^+^ cytotoxic cells cause cell destruction, leading to the release of nucleic acids and autoantigens. These complexes can stimulate antigen-presenting cells, promote T cell activation, recruit lymphocytes, and enhance the development of effector cells.^84^, ^85^, ^86^, ^87^ Research indicates that in patients with AA, the progression of HF damage is largely influenced by the presence of inflammatory cells, including CD4^+^ and CD8^+^ T cells, surrounding and infiltrating HFs.^88^ Significant cell death during inflammation often results from the close interaction between lymphocytes and target cells, with CD8^+^ T cells having a greater ability to penetrate intra-follicular areas compared with CD4^+^ T cells. The identification of HF autoantigens recognized by the T cell receptors of autoreactive CD8^+^ cytotoxic T cells is now considered crucial.^14^^,^^89^ Increasing evidence suggests that CD8^+^ T lymphocytes are key contributors to the development and persistence of alopecia disorders.^90^ In mouse models, the elimination of CD4^+^ or CD8^+^ T cells through monoclonal antibodies leads to hair regrowth.^91^^,^^92^ Subcutaneous injection of CD8^+^ T cells results in localized hair loss, while CD4^+^ T cell injection causes systemic AA, highlighting the distinct roles of these two T cell types in skin disorders.^93^^,^^94^ Studies have shown that NKG2D is significantly expressed in the HF cells of AA patients, where CD8^+^ T cells are localized.^95^ Single-cell sequencing has revealed CD8^+^ T cell clusters expressing NKG2D in the skin and skin-draining lymph nodes of alopecia mice, along with other genes associated with natural killer cells, such as NKG7. This gene expression pattern mirrors the CD8^+^ T cell clusters observed in AA patients, particularly in affected skin, where NKG7 is also present.^96^ CD8^+^ T cells may damage HF cells through direct mechanisms involving the Fas or perforin pathway, or indirectly by producing cytokines, leading to cell and tissue destruction.^97^ Apoptosis is significantly elevated in inflamed areas, and the pathophysiology of HFs is severely altered, although the follicles are not completely destroyed.^98^ Due to the strong regenerative ability of HFs, any residual IP activity may partially inhibit the effects of CD8^+^ T cells.^99^

## The key regulatory genes on T cells in hair regeneration

In recent years, an increasing number of studies have highlighted the significance of immune regulation in HFs. Consequently, there is a pressing need for further investigation into the differentiation and maturation of T cells. The differentiation of T cells is influenced by various factors, including cytokines, transcription factors, signaling pathways, and microRNAs (Table 2).^100^ We have summarized the following four aspects to review the key mechanisms that regulate T cell differentiation and development.

## Cytokines

Cytokines play a crucial role in influencing the differentiation and plasticity of various CD4^+^ T cell subsets. For instance, IL-6 is essential for enhancing the production of RORγt and facilitating the development of Th17 cells through the activation of the JAK2-STAT3 signaling pathway.^101^ IL-1β promotes Th17 polarization via the IL-1R/PI3K-mTOR signaling pathway and suppresses Foxp3 expression in CD4^+^ T cells induced by TGF-β, thereby hindering Treg differentiation. ^102^^,^^103^ IL-4, produced by Th2 and natural killer T cells, stimulates the proliferation of activated T and B cells and influences the development of both Th1 and Th2 cells.^104^ Meanwhile, IL-12 induces the production of T-bet and IFN-γ in T cells, key Th1 transcription factors generated by the TBX21 gene, via STAT4 signaling activation.^105^ Additionally, IL-21, produced by Th17 cells, enhances the levels of IL-17 and RORγt, promoting Th17 cell development through the STAT3 signaling pathway.^106^ IL-23 may further support the persistence and growth of Th17 cells without altering their differentiation.^107^ CD4^+^ T cells can differentiate into Tregs in the presence of high levels of TGF-β; conversely, when TGF-β is combined with low levels of IL-6, they differentiate into Th17 cells, activating Smad2 and promoting the production of IL-22.^108^^,^^109^

Moreover, studies indicate that the release of cytokines significantly influences lymphocyte infiltration in HFs and disrupts the hair growth cycle. An imbalance in cytokine expression is one of the key causes of hair diseases.^110^ For example, IL-6 may contribute to wound-induced HFs by activating STAT3, while vascular endothelial growth factor enhances the formation of new blood vessels surrounding HFs. Epidermal growth factor boosts the function and proliferation of outer root sheath cells in HFs by stimulating Wnt/β-catenin signaling. Bone morphogenetic protein (BMP) helps maintain the dermal papilla cell phenotype, and platelet-derived growth factor can accelerate the transition of HFs from the resting stage to the growing stage.^111^

Therefore, further elucidation of the pathogenic roles and clinical significance of cytokines may provide valuable insights for developing new therapeutic strategies for HFs.

## Transcription factors

Transcription factors (TFs) are pivotal in regulating CD4^+^ T cell differentiation. For instance, HIF-1α serves as a nuclear TF, with its expression up-regulated in response to stimuli such as TNF-α and PI3K, thereby regulating the stability and function of immune cells.^100^ HIF-1α can specifically trigger the release of IL-12p40, which inhibits the transformation of Th0 cells into Th1 cells.^112^ Additionally, evidence suggests that HIF-1α is crucial for regulating Th17 cell activity and IL-17 expression.^113^ In the absence of HIF-1α, the differentiation of Th0 cells into Th17 cells is impeded, and the production of RORγt is diminished.^114^ Moreover, other TFs, such as T-bet, GATA3, RORγt, and Foxp3, also play significant roles in T cell differentiation.^115^ T-bet establishes a positive feedback loop with the STAT1 signaling axis, promoting Th1 polarization by up-regulating IL-12.^116^ GATA3, a T-cell-specific TF, is a central regulatory factor for maintaining the efficacy of Th2 responses.^117^^,^^118^ Foxp3 is the principal TF governing the development and function of Tregs; its up-regulation, alongside the down-regulation of RORγt, inhibits Th17 cell differentiation and restores Treg functionality.^118^ As highlighted, TFs are foundational to controlling selective immune responses. Given the relationship between T cells and HF regeneration, TFs are also critical factors influencing the development and maintenance of HFSCs. Thus, they represent one of the most promising yet underutilized targets for therapeutic interventions.^119^

## Signal pathways and key signal molecules

Signal transduction pathways are vital communication routes that facilitate interactions between a cell's internal and external environments, playing a crucial role in the differentiation and development of T cells. Several evolutionarily conserved pathways, including Notch, BMP, Hedgehog, FGF, TGF-β, and Wnt signaling, regulate the formation and maintenance of adult stem cells.^120^ For instance, TGF-β functions as a pleiotropic factor in controlling immune responses, promoting the differentiation of CD4^+^ T cells into Th9, Th17, Treg, and Tfh cells while inhibiting the formation of Th1 and Th2 cells. Research indicates that elevated levels of pro-inflammatory factors such as IL-6, IL-17, and TNF-α decrease, whereas the expression of the anti-inflammatory factor IL-10 and the Treg-specific transcription factor Foxp3 increase, resulting in a reduced Th17/Treg cell ratio. IL-27 can inhibit Treg differentiation by blocking TGF-β′s ability to induce Foxp3 expression. Additionally, STAT3 may play a role in this process, independent of IL-27's inhibitory effects on IL-2, which is essential for Treg differentiation. TGF-β may also be present in dendritic cells via an alternative NF-κB pathway, fostering immunological tolerance and suppressing T cell development and proliferation. TGF-β1 has been shown to strongly inhibit pro-inflammatory factors such as TNF-α and IFN-γ, while the proliferation of CD4^+^ T cells is influenced by the costimulatory protein CD28. Specifically, TGF-β1 suppresses the growth of inactive T cells lacking CD28 but promotes the growth and specialization of CD28-positive T cells.^121^

Additionally, Smad4, a member of the Smad signal transduction family, is crucial in the TGF-β signaling pathway, influencing HF growth and differentiation. The TGF-β/BMP signaling pathway can determine the growth stage of HFs through a negative feedback mechanism. The Hedgehog signaling pathway differentiates developing T cells into MHC-restricted, autoantigen-tolerant T cells in the thymus in a concentration-dependent manner and is also associated with hair growth. The classical Wnt signaling pathway is essential for thymus development and T lymphocyte differentiation and maturation. Studies show that increased canonical Wnt signaling, driven by stabilized β-catenin, leads to decreased FOXN1 expression, which hinders T lymphopoiesis and results in T cell hypoplasia.^122^ Moreover, the Wnt/β-catenin signaling system plays a crucial role in all phases of HF development, influencing cell differentiation throughout growth.^123^ HFSCs are regulated by CD4^+^ T cells and macrophages during the resting phase, with the HF bulge communicating with immune cells recruited to counteract signals from the resting stage. Thus, the precise management of these intricate communication pathways is vital for the differentiation and development of T lymphocytes and the regulation of HFSC proliferation and differentiation.^124^

## MicroRNAs

MicroRNAs are key components of an epigenetic process that can induce inheritable changes in phenotype without altering the DNA sequence. Disturbances in the gene expression patterns regulated by epigenetics can lead to autoimmune disorders, malignancies, and other diseases.^125^ MicroRNAs play a crucial role in regulating the immune response by facilitating the development and activation of various immune cells, including B and T lymphocytes, macrophages, and dendritic cells.^126^ The expression patterns of microRNAs vary depending on the tissue and remain consistent across different clinical conditions, making the regulation of microRNAs a promising therapeutic target.^126^ Several studies have indicated that abnormal expression and function of microRNAs are associated with the pathogenesis, progression, maintenance, or exacerbation of skin conditions such as HS, LLP, and atopic dermatitis.^127^ Increased levels of miR-21 are linked to elevated IL-22 expression, underscoring its role in sustaining skin inflammation and T cell activation and promoting keratinocyte growth by influencing the STAT3 and NF-κB signaling pathways. Suppressing miR-21 has been shown to reduce the levels of Th17-associated cytokines, including IL-17A, IL-17F, IL-21, IL-22, and TNF-α. Altered expression of miR-146a in HS patients affects the TRAF6/IRAK-1 pathways, leading to decreased TNF-α production.^77^ Similarly, elevated miR-155 expression contributes to increased production of pro-inflammatory cytokines (*e.g.*, IL-6, IL-1β, IL-8, TNF-α) and influences the development and activity of Th17 cells.^77^ miR-125b regulates the development and proliferation of keratinocytes by inhibiting fibroblast growth factor receptor 2 (FGFR2).^77^ Additionally, potential target genes of miR-338 include RNF114, TNFR1, TRADD, and RUNX1. RNF114 acts as a suppressor of T cell activation and is involved in T cell apoptosis.^128^ Overexpression of miR-338 in CD4^+^ T cells results in decreased levels of the transcription factor Foxp3, leading to Treg dysfunction due to its impact on RUNX1.^128^^,^^129^

In summary, specific microRNAs, including miR-21, miR-146a, miR-155, miR-125b, and miR-338, significantly influence the activity and differentiation of various immune cells, particularly T cells and keratinocytes. These microRNAs modulate critical signaling pathways and cytokine production, such as STAT3, NF-κB, and TRAF6/IRAK-1, which are essential for maintaining the balance between pro-inflammatory and anti-inflammatory responses in the skin. By affecting the immune environment surrounding HFs, microRNAs play a significant role in processes that can either promote or hinder HF regeneration and growth, as detailed in Table 2.

## Conclusions

The skin serves as the primary habitat for diverse immune cells due to its constant exposure to environmental pathogens. HFs are formed by indentations in the outer layer of the skin, where symbiotic bacteria reside and function as the first barrier against harmful germs. Lymphocytes, myeloid cells, and epithelial cells collaborate around the HFs to maintain the skin's barrier function. These skin-dwelling immune cells undergo dynamic changes during hair cycle development, indicating their active role in regulating HF regeneration.^130^ A notable connection between the changes in skin immune cells throughout the hair cycle and hair regrowth is illustrated by the observation that allograft rejection occurs more slowly when HFs in the donor skin are in the resting phase rather than the growth phase.^131^ The intricate regulatory mechanisms within the HF regeneration cycle ensure proper synthesis and anchoring of hairs in the skin, fulfilling their biological functions, including thermoregulation, communication, and sensory perception. Hepatocyte growth factors interact with various cells to initiate a regenerative cycle.^132^ Importantly, HFs are not merely passive receivers of immunogenic signals; rather, immune cells are recruited to the parafollicular region, creating an immunological environment conducive to regeneration.^133^ Additionally, HF-derived IL-17 and IL-15 are crucial for maintaining CD4^+^ T and CD8^+^ T cell populations within the skin.^133^

In conclusion, substantial evidence supports the role of T cell-mediated immune microenvironment homeostasis in HFs. The HF system offers a valuable opportunity to explore the relationship between stem cells and their surrounding environments. This regeneration process is meticulously regulated both temporally and spatially, enabling the observation of alterations in hair development without causing harm to animals.

## Prospects

With the increasing demands of society and rising work-life pressures, hair loss has become a common and increasingly serious issue. Currently, minoxidil and finasteride are the first-line treatments for alopecia, but their use is often associated with serious adverse reactions, and the risk of recurrence is high after withdrawal.^134^ The HF microenvironment regulates the HF cycle, and disruptions in this microenvironment often lead to impaired homeostasis and regeneration of HFs.^135^, ^136^, ^137^ The success of immunomodulation in restoring hair growth in patients with AA highlights the potential of immunomodulatory therapies as a promising area of research.^5^ Among these, JAK inhibitors have emerged as one of the most representative approaches.^138^ In addition, immunosuppressants like cyclosporine, which inhibit T cell activation, and corticosteroids, which reduce immune activity around HFs, are also used.^139^^,^^140^ Although recent advances have deepened our understanding of the relationship between HFs and immune system regulation, several intriguing challenges remain.^130^ Notably, research on immune-mediated HFs is at a pivotal stage, where the complexity of the microenvironment poses significant challenges. For example, mouse hair does not accurately reflect human hair growth patterns, signal pathways, or immunogenicity. Many molecular mechanisms underlying this relationship have not yet been confirmed in humans, blending basic scientific research with clinical medicine.^132^ Looking ahead, it will be important to investigate the regulatory effects of different immune cells near HFs, assess their variability across different skin regions, and study their responses under various physiological and pathological conditions.^130^ Understanding the connection between the immune system and HFs could enhance medication delivery methods and treatment efficacy. Moreover, advances in genetic research are opening up possibilities for personalized medicine, where treatments could be tailored to an individual's unique genetic makeup, immune system irregularities, and disease symptoms.^141^ This approach, focusing on precise medical treatments tailored to a patient's biological fingerprint, holds great promise for improving treatment outcomes for alopecia and other germinal disorders. To maximize therapeutic success, a broader understanding of the immunological underpinnings of hair loss is essential.

## Funding

This work was supported by the Research Project of Zhejiang Chinese Medical University (No. 2024FSYYZZ09), Zhejiang Provincial Department of Education General Scientific Research Project (No. Y202351272) and the Opening Fund of the 10.13039/501100015249State Key Laboratory of Quality Research in Chinese Medicine, University of Macau, Macau, China (No. QRCM-OP21002). The funders had no role in study design; in the collection, analysis, and interpretation of data; in the writing of the report; and in the decision to submit the article for publication.

## CRediT authorship contribution statement

**Nana Tao:** Writing – original draft. **Qingru Sun:** Writing – review & editing. **Yuyuan Ying:** Writing – review & editing. **Yitao Wang:** Writing – review & editing. **Jianli Gao:** Writing – review & editing.

## Conflict of interests

The authors declared no competing interests.
